# Validation of a tool for estimating clinician recognition of ARDS using data from the international LUNG SAFE study

**DOI:** 10.1371/journal.pdig.0000325

**Published:** 2023-08-25

**Authors:** Meagan A. Bechel, Fabiana Madotto, Adam R. Pah, Giacomo Bellani, John G. Laffey, Tài Pham, Luís A. Nunes Amaral, Curtis H. Weiss

**Affiliations:** 1 Department of Radiology and Imaging Sciences, Emory University, Atlanta, Georgia, United States of America; 2 IRCCS Multimedica, Value-based Healthcare Unit, Sesto San Giovanni, Milan, Italy; 3 Kellogg School of Management, Northwestern University, Evanston, Illinois, United States of America; 4 ASST Monza, Sam Gerardo Hospital and Department of Medicine and Surgery, University of Milan-Bicocca, Monza, Italy; 5 School of Medicine, National University of Ireland Galway, Galway, Ireland; 6 Service de médecine intensive-réanimation, AP-HP, Hôpital de Bicêtre, Hôpitaux Universitaires Paris-Saclay, Le Kremlin-Bicêtre, France; 7 Université Paris-Saclay, UVSQ, Univ. Paris-Sud, Inserm U1018, Equipe d’Epidémiologie respiratoire intégrative, CESP, 94807, Villejuif, France; 8 Department of Chemical and Biological Engineering, Northwestern University, Evanston, Illinois, United States of America; 9 Northwestern Institute on Complex Systems, Northwestern University, Evanston, Illinois, United States of America; 10 Department of Medicine, Northwestern University, Chicago, Illinois, United States of America; 11 Department of Medicine, NorthShore University HealthSystem, Evanston, Illinois, United States of America; Yonsei University College of Medicine, REPUBLIC OF KOREA

## Abstract

Under-recognition of acute respiratory distress syndrome (ARDS) by clinicians is an important barrier to adoption of evidence-based practices such as low tidal volume ventilation. The burden created by the COVID-19 pandemic makes it even more critical to develop scalable data-driven tools to improve ARDS recognition. The objective of this study was to validate a tool for accurately estimating clinician ARDS recognition rates using discrete clinical characteristics easily available in electronic health records. We conducted a secondary analysis of 2,705 ARDS and 1,261 non-ARDS hypoxemic patients in the international LUNG SAFE cohort. The primary outcome was validation of a tool that estimates clinician ARDS recognition rates from health record data. Secondary outcomes included the relative impact of clinical characteristics on tidal volume delivery and clinician documentation of ARDS. In both ARDS and non-ARDS patients, greater height was associated with lower standardized tidal volume (mL/kg PBW) (ARDS: adjusted β = -4.1, 95% CI -4.5 –-3.6; non-ARDS: β = -7.7, 95% CI -8.8 –-6.7, *P*<0.00009 [where α = 0.01/111 with the Bonferroni correction]). Standardized tidal volume has already been normalized for patient height, and furthermore, height was not associated with clinician documentation of ARDS. Worsening hypoxemia was associated with both increased clinician documentation of ARDS (β = -0.074, 95% CI -0.093 –-0.056, *P*<0.00009) and lower standardized tidal volume (β = 1.3, 95% CI 0.94–1.6, *P*<0.00009) in ARDS patients. Increasing chest imaging opacities, plateau pressure, and clinician documentation of ARDS also were associated with lower tidal volume in ARDS patients. Our EHR-based data-driven approach using height, gender, ARDS documentation, and lowest standardized tidal volume yielded estimates of clinician ARDS recognition rates of 54% for mild, 63% for moderate, and 73% for severe ARDS. Our tool replicated clinician-reported ARDS recognition in the LUNG SAFE study, enabling the identification of ARDS patients at high risk of being unrecognized. Our approach can be generalized to other conditions for which there is a need to increase adoption of evidence-based care.

## Author summary

Under-recognition of acute respiratory distress syndrome (ARDS) by clinicians has been the primary barrier to the adoption of evidence-based practices such as low tidal volume ventilation. In a secondary analysis of the international LUNG SAFE study that included 2,705 ARDS patients and a novel cohort of 1,261 non-ARDS patients, a trained ARDS recognition tool accurately replicated clinician-reported ARDS recognition using patient height, gender, and lowest tidal volume, characteristics readily available in the electronic health record. Greater height was associated with lower tidal volume in both ARDS and non-ARDS patients, and was more predictive of tidal volume than the presence of ARDS itself. This study demonstrates that a data-driven ARDS recognition tool can identify patients at high risk of clinician under-recognition while accounting for influential patient characteristics such as height.

## Introduction

Severe coronavirus disease 2019 (COVID-19) principally manifests as acute respiratory distress syndrome (ARDS), albeit potentially as a unique phenotype. [1–4] ARDS is a heterogeneous syndrome characterized by acute hypoxemic respiratory failure and non-cardiogenic pulmonary edema that results from inflammatory lung injury and diffuse alveolar damage. [5] ARDS is usually associated with an identifiable clinical risk factor, such as pneumonia or sepsis. [5–6] In pre-COVID-19 studies, ARDS affected 10.4% of patients admitted to the intensive care unit (ICU) worldwide and was associated with a mortality rate of 27–46%. [5–6] Low tidal volume ventilation (LTVV)—a ventilator strategy of limiting tidal volume and plateau pressure (P_plat_) to prevent further lung injury—reduces mortality in ARDS patients by almost 25% and is a strongly recommended practice. [7–8] However, LTVV utilization is believed to be as low as 19%. [6,9–10]

Clinician under-recognition of ARDS is a major barrier to LTVV. [6,11] Previously, we piloted a computational method to estimate clinician ARDS recognition using characteristics readily available in the electronic health record (EHR), including patient height, gender, and tidal volume, thus reducing potential bias and resource needs. [11]

Here, we advance our prior work and validate this EHR-based data-driven tool for estimating clinician ARDS recognition through a secondary analysis of the Large Observational Study to Understand the Global Impact of Severe Acute Respiratory Failure (LUNG SAFE) cohort, the largest epidemiological study of ARDS to date. [6] Based on our prior work, we hypothesized that a recognition estimation tool trained on data readily available in the EHR would yield clinician ARDS recognition rates similar to self-reported ARDS recognition rates collected from LUNG SAFE clinicians. With the significant changes in practice and burdens imposed by the COVID-19 pandemic, low-burden data-driven approaches that help institutions assess and improve recognition of ARDS by critical care physicians have become indispensable.

## Materials and methods

### Data collection

This is a secondary analysis of the LUNG SAFE study, a prospective cohort study of ARDS incidence in 459 ICUs across 50 countries. [6] In the LUNG SAFE study, ARDS cohort status was assigned when, within the same 24-hour period, all Berlin Definition criteria were present via the case report form (CRF) or electronic health record auditing. [5] Separately, in the original LUNG SAFE study, the clinician was deemed to have recognized ARDS in a patient if they selected ARDS as a diagnosis on at least one of two separate CRFs. [6]

**Fig 1:**
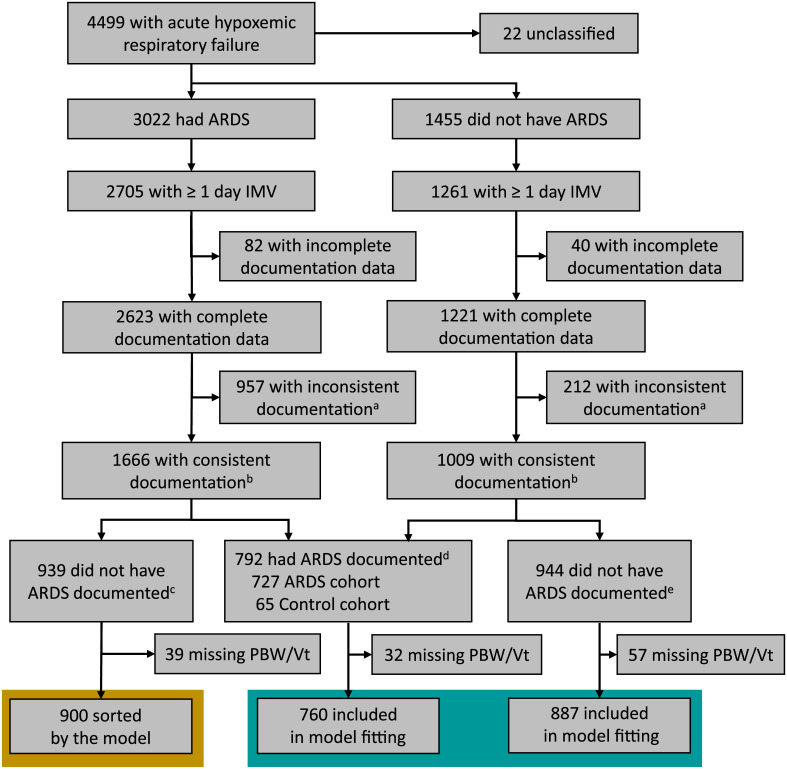
Inclusion criteria and data flow. ^a^ If the subject’s reported ARDS status changed between study entry and end (Yes/No, No/Yes). ^b^ If the subject’s reported ARDS status did not change between study entry and end (Yes/Yes, No/No). ^c^ ARDS non-documented subgroup. ^d^ Pooled documented subgroup. ^e^ Control non-documented subgroup. ARDS: acute respiratory distress syndrome, IMV: invasive mechanical ventilation, PBW: predicted body weight, Vt: tidal volume.

LUNG SAFE identified 3,022 patients who met the Berlin Definition of ARDS during four-week winter periods in 2014. In this study, we included the 2,705 patients diagnosed with ARDS who received invasive mechanical ventilation, similar to our previous inclusion criteria (Fig 1). [11]

Additionally, we established a non-ARDS “control” group by selecting the LUNG SAFE patients who had acute hypoxemic respiratory failure (P_a_O_2_/F_I_O_2_≤300mm Hg and new pulmonary parenchymal opacities) and who received invasive mechanical ventilation, but were determined not to have ARDS (n = 1,261, Fig 1).

Site clinicians were asked whether a patient had ARDS on separate CRFs on day 1 (study entry) and at ICU discharge or death (study end). At study entry, clinicians selected all potential causes of hypoxemia from a list that included ARDS. At study end, clinicians were asked explicitly if the patient ever had ARDS during their hospital course. [6]

All study variables (P_a_O_2_/F_I_O_2_, tidal volume [mL], other ventilator settings and measurements, and clinical characteristics) were recorded on multiple days. We defined standardized tidal volume as ${\hat{\text{V}}}_{\text{T}}={\text{V}}_{\text{T}}/\text{PBW}$ (mL/kg). Data availability for each variable is listed in S1 and S2 Tables. We collected other clinical and outcomes data as previously described. [6]

This study was determined exempt by the Northwestern University Institutional Review Board (STU00205441) and was approved by the OPEN-LUNG-SAFE initiative.

### Recognition tool assumptions

Our approach relies on the following two assumptions:

1. Tidal volumes delivered in both ARDS and non-ARDS patients are influenced by clinical characteristics.
2. Physicians deliver lower standardized tidal volumes to patients they recognize as having ARDS than they would otherwise. [12–13]
  These assumptions suggest that delivered standardized tidal volume provides a proxy for clinician recognition of ARDS. Further, they suggest a corollary:
3. The ARDS cohort can be viewed as a mixture of recognized and unrecognized patients.

The goal of our approach is to develop an automated data-driven tool that determines whether a patient with ARDS was recognized by clinicians or not. It is important to note that the goal of this tool is not to predict whether a patient has or does not have clinical ARDS (an independent variable determined by chart review) but rather whether a clinician makes a diagnosis of ARDS for a patient. To accomplish this goal, first we separately evaluated the impact of clinical characteristics on tidal volume delivery in ARDS and non-ARDS patients (assumption #1; cohorts defined by chart review).

### Clinical characteristics

To determine which factors should be accounted for in our clinician ARDS recognition tool, we employed ordinary least squares (OLS) regression to assess associations between these factors and lowest ${\hat{\text{V}}}_{\text{T}}$ (S1 Text).

We evaluated significant univariable factors for covariance with each other. We excluded from our analysis pairs of factors where the difference in data availability (S1 and.S2 Tables) between the two factors for the cohort being considered exceeded 10% of cohort size.

We constructed multivariable regressions using all combinations of the significant factors from the univariable analysis and appropriate interaction terms, excluding some factors due to covariance as described above. We calculated AIC (Akaike Information Criterion) and BIC (Bayesian Information Criterion) for each regression to select the “best” model fit. We used Python and the package *statsmodels* (version 0.6.1) for all calculations.

We report results from regression analyses as normalized β-coefficients with 95% confidence intervals (CI).

#### Significance testing

We used α = 0.01 and applied the Bonferroni correction for multiple hypotheses. In the univariable analyses, there were 111 comparisons where lowest ${\hat{\text{V}}}_{\text{T}}$ was the dependent variable, thus we set *P*<0.00009 (0.01/111) as the threshold for statistical significance for these analyses. For the covariate analyses, the threshold was *P*<0.001 (0.01/10).

### Sensitivity analyses

In our pilot study, we defined clinician documentation of ARDS as the presence of an ARDS diagnosis in a note written by an attending physician. To replicate this strict definition as closely as possible without access to clinician notes, we defined clinician documentation of ARDS in the LUNG SAFE data as the presence of affirmative identification of ARDS as a diagnosis on *both* the study entry and study end CRFs, which differs from the original LUNG SAFE definition. This definition of documentation will be inaccurate for the case of patients who developed ARDS after study entry (e.g., day 2 or 3). In this case, a patient could meet Berlin Definition criteria on day 2 or 3 but the clinician would not have been incorrect on the study entry CRF since the patient did not have ARDS at that time. We believe this is not a significant limitation for three reasons. First, we conducted a sensitivity analysis using only the study end CRF documentation and obtained similar results (S3 Table). Second, only 12% of patients developed ARDS after day 1. Third, using the strict definition of documentation (both entry and end CRFs) allows us to be reassured that clinicians truly recognized ARDS. Since we do not know who was responsible for filling entry and end CRF, we cannot be as confident that ARDS was clearly identified by a clinician if only one form included ARDS documentation.

As an additional sensitivity analysis, we repeated all analyses when restricting the cohort to only patients that were on the assist control ventilator mode on the majority of days (n = 409 for ARDS cohort, n = 213 for control cohort).

### Tool construction

Guided by our above assumptions, we divided the ARDS and control cohorts into three groups (Fig 1): [11]

1. ARDS non-documented: ARDS patients where the site clinicians did not document ARDS on both forms,
2. control non-documented: non-ARDS patients where the site clinicians did not document ARDS on both forms, and
3. pooled documented: patients from both ARDS and non-ARDS cohorts where the site clinicians documented ARDS on both forms.

Our tool classifies patients as either “recognized” or “unrecognized.” All patients in the pooled documented group were classified as “recognized.” Patients in the ARDS non-documented group were classified according to a naïve Bayes model that determined whether the lowest standardized tidal volume they received was most similar to the lowest standardized tidal volume delivery seen in the pooled documented group or the lowest standardized tidal volume delivery seen in the control non-documented group. Specifically, leveraging Bayes Theorem, we classified a patient in the ARDS non-documented subgroup as recognized or unrecognized based on the following conditional probabilities:

$$\frac{P(documented|X,{\hat{V}}_{T})}{P(control|X,{\hat{V}}_{T})}=\frac{P\left(X,{\hat{V}}_{T}|documented\right)*P(documented)}{P\left(X,{\hat{V}}_{T}|control\right)*P(control)}$$

In the absence of a better supported prior for *P(documented)* and *P(control)*, we set them to 0.5; that is, we assume equal *a priori* probability of belonging to either subgroup. X represents the most influential factor, i.e., highest standardized beta coefficient in the regression analysis of ${\hat{\text{V}}}_{\text{T}}$ in both the pooled documented and control non-documented subgroups.

This approach enables us to determine a boundary in the X vs. lowest ${\hat{\text{V}}}_{\text{T}}$ plane defined by the equality $P(documented|X,{\hat{V}}_{T})=P(control|X,{\hat{V}}_{\text{T}})$, using kernel density estimation to estimate the probability of observing a specific pair of values of lowest ${\hat{\text{V}}}_{\text{T}}$ and X for both the pooled documented and control non-documented subgroups (Fig 1). Due to the size discrepancy between the control non-documented and pooled documented subgroups, we bootstrapped (100 iterations) the control non-documented subgroup and repeated the analysis to produce confidence bands from the placement of the boundary (S1 Fig). For patients with lowest ${\hat{\text{V}}}_{\text{T}}\text{s}$ below this boundary, $P(documented|X,{\hat{V}}_{T})$ is greater than *$P(control|X,{\hat{V}}_{\text{T}})$* and the patient is classified as “recognized.” Patients with lowest ${\hat{\text{V}}}_{\text{T}}\text{s}$ above this boundary are classified as “unrecognized.”

## Results

Of the 2,705 ARDS patients and 1,261 non-ARDS control patients from the LUNG SAFE study that we included, 792 patients were documented by clinicians as having ARDS on both the entry and exit CRFs; 92% of them were in the LUNG SAFE ARDS cohort (Fig 1).

### Clinical characteristics

#### Predictors of Tidal Volume Use—Univariable Analysis

**Fig 2:**
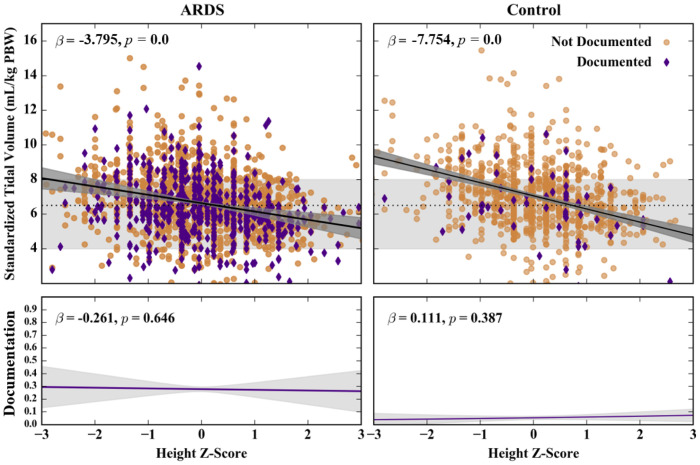
Effects of patient height on standardized tidal volume (${\hat{\text{V}}}_{\text{T}}$) and ARDS documentation in ARDS and control cohorts. Top panels show patients with ARDS documented (purple diamonds) and non-documented (tan circles). Gray areas represent LTVV range from current guidelines, with dashed line at 6.5 mL/kg PBW. Solid lines show linear (${\hat{\text{V}}}_{\text{T}}$) and logistic (documentation) fits for scatter plot data (shaded regions, 95% confidence bands). Reported beta coefficients are for standardized inputs. ARDS: acute respiratory distress syndrome, LTVV: low tidal volume ventilation, PBW: predicted body weight, ${\hat{\text{V}}}_{\text{T}}$: standardized tidal volume (mL/kg PBW).

Lowest ${\hat{\text{V}}}_{\text{T}}$ decreased as patient height z-score increased in both the ARDS and control cohorts (ARDS: β-coefficient, -3.8, 95% CI, [-4.2, -3.4], *P*<0.00009; control: β-coefficient, -7.7, 95% CI, [-8.8, -6.7], *P*<0.00009; Table 1, Fig 2). In addition, in the ARDS cohort, lowest ${\hat{\text{V}}}_{\text{T}}$ decreased with worsening ARDS severity, increasing number of chest X-ray quadrants with infiltrates, higher P_plat_, and clinician documentation of ARDS (*P*<0.00009 for each characteristic, Table 1, S2 Fig). These characteristics were not associated with lower ${\hat{\text{V}}}_{\text{T}}$ in the control cohort. SOFA score, ICU admission weight, study region, patient study enrollment age, and ventilator mode were also not associated with lower ${\hat{\text{V}}}_{\text{T}}$ in either the ARDS or control cohorts.

**Table 1:** Predictors of lowest ${\hat{\text{V}}}_{\text{T}}$ (mL/kg PBW) (β-coefficient [95% CI]).

Factor	ARDS		Control univariable	Documented Univariable^a^
	univariable	multivariable		
Height Z Score	**-3.8** ^b^ **[-4.2,-3.4]**	**-3.7** ^b^ **[-4.3, -3.2]**	**-7.7** ^b^ **[-8.8, -6.7]**	**-3.7** ^b^ **[-4.4, -3.0]**
P_a_O_2_/F_I_O_2_ ratio				
Entry	**0.64** ^b^ **[0.35, 0.93]**		0.26 [-0.23, 0.75]	0.72 [0.18, 1.3]
End	0.10 [-0.38, 0.58]		-0.58 [-1.5, 0.31]	-0.40 [-1.2, 0.37]
Lowest	**1.3** ^b^ **[0.94, 1.6]**	**1.2** ^b^ **[0.90, 1.6]**	0.54 [0.02, 1.1]	**1.4** ^b^ **[0.81, 2.0]**
Documentation				
Entry	**-0.39** ^b^ **[-0.54, -0.24]**		-0.12 [-0.57, 0.33]	
End	**-0.41** ^b^ **[-0.56, -0.26]**		-0.43 [-0.72, -0.14]	
Both	**-0.39** ^b^ **[-0.55, -0.23]**	-0.31 [-0.46, -0.15]	-0.59 [-1.1, -0.1]	
P_plat_				
Entry	-1.0 [-1.7, -0.34]		-0.28 [-1.1, 0.58]	-0.54 [-1.5, 0.40]
End	-1.2 [-1.9, -0.47]		-0.57 [-1.5, 0.39]	-0.8 [-1.6, 0.084]
Highest	**-1.5** ^b^ **[-2.3, -0.87]**		-1.1 [-2.1, -0.19]	-1.2 [-2.1, -0.25]
Chest imaging quadrants				
Entry	**-0.49** ^b^ **[-0.65, -0.33]**		-0.42 [-0.79, -0.05]	-0.79 [-1.2, -0.36]
End	**-0.69** ^b^ **[-1.0, -0.39]**		-0.56 [-1.2, 0.05]	-0.43 [-0.96, 0.11]
Highest	**-1.4** ^b^ **[-1.7, -1.1]**		-0.77 [-1.2, -0.36]	**-1.5** ^b^ **[-2.1,-0.86]**
SOFA				
Entry	0.1 [-0.44, 0.64]		0.21 [-0.53, 0.96]	0.57 [-0.31, 1.5]
End	0.0 [-0.53, 0.58]		0.95 [0.04, 1.9]	0.52 [-0.43, 1.5]
Highest	-0.39 [-0.88, 0.09]		0.25 [-0.52, 1.0]	0.23 [-0.6, 1.1]
ICU admission weight	-1.2 [-2.0, -0.51]		-0.52 [-1.8, 0.78]	-2.7 [-4.1, -1.3]
Study Age	-0.38 [-1.3, 0.56]			0.29 [-0.89, 1.5]
Region	-0.8 [-1.1, -0.52]		1.7 [0.86, 2.5]	
Modality	0.18 [-0.15, 0.50]		0.02 [-0.53, 0.56]	-0.51 [-1.1, 0.13]

^*a*^ Documentation on both study entry and exit forms.

^*b*^ *P*<0.00009. Empty cells indicate category was not used due to data being unavailable or not relevant. ARDS: acute respiratory distress syndrome. PBW: predicted body weight. P_plat_: Plateau airway pressure (cm H_2_O). SOFA: Sequential Organ Failure Assessment. ${\hat{\text{V}}}_{\text{T}}$: Standardized tidal volume (mL/kg PBW).

In the pooled documented subgroup, the factors associated with lower ${\hat{\text{V}}}_{\text{T}}$ included height z-score, ARDS severity, and number of chest X-ray quadrants with infiltrates (Table 1). Additional associations between clinical characteristics and lowest ${\hat{\text{V}}}_{\text{T}}$ in the documentation subgroups can be found in S3 and S4 Tables.

#### Predictors of Tidal Volume Use—Covariate Analysis

As logic demands, height was not associated with clinician documentation of ARDS in either the ARDS or control cohorts (Fig 2). However, worsening hypoxemia severity was associated with increased clinician documentation of ARDS in the ARDS cohort (S2 Fig).

Lowest P_a_O_2_/F_I_O_2_, highest number of chest X-ray quadrants with infiltrates, chest X-ray quadrants with infiltrates at study end, highest P_plat_, and clinician documentation of ARDS at study end were covariate with each other (*P*<0.00009, S5 Table). ARDS documentation at study entry was correlated with number of chest X-ray quadrants with infiltrates at study entry. Patient height was not associated with any severity markers.

#### Predictors of Tidal Volume Use—Multivariable analysis

Out of the seven multivariable regressions constructed for the ARDS cohort, the regression including height z-score, lowest P_a_O_2_/F_I_O_2_, and clinician ARDS documentation as independent variables and a lowest P_a_O_2_/F_I_O_2_:documentation interaction term resulted in the best fit (S6 Table). Multivariable analysis was not performed for the control cohort since height z-score was the only characteristic associated with lowest ${\hat{\text{V}}}_{\text{T}}$.

In the best fit multivariable regression, height z-score and lowest P_a_O_2_/F_I_O_2_ remained predictors of lowest ${\hat{\text{V}}}_{\text{T}}$ (*P*<0.00009, Table 1). Of these variables, height z-score was the strongest predictor of lowest ${\hat{\text{V}}}_{\text{T}}$ (β-coefficient, -3.7, 95% CI, [-4.3, -3.3], *P*<0.00009).

As a sensitivity analysis, we restricted the cohorts to only patients that were on the assist control/volume control mode of ventilation and repeated the above analyses. Except for the study end documentation subgroup, the only factor significantly associated with ${\hat{\text{V}}}_{\text{T}}$ was height z-score in all other cohorts and subgroups (S7 Table). In the study end documentation subgroup, height z-score and highest number of chest x-ray quadrants with infiltrates were correlated with ${\hat{\text{V}}}_{\text{T}}$. Given that the final tool only requires the most influential factor, which was height z-score in all subgroups, neither restriction of ventilator mode nor relaxation of the ARDS recognition definition had an impact on tool construction.

### Tool for estimating ARDS Recognition rate

**Fig 3:**
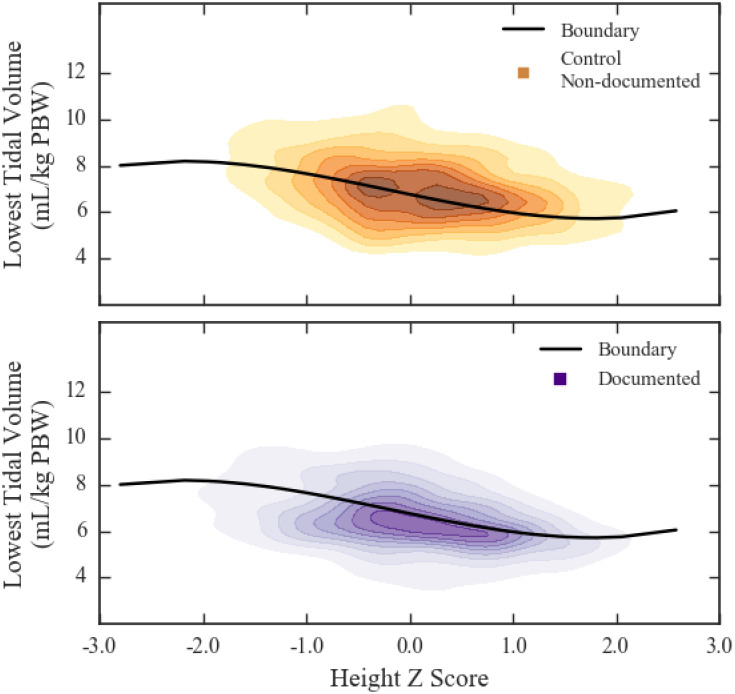
Kernel Density Estimation for control non-documented and pooled documented patients. Heatmaps of kernel density estimated probability density for data from control non-documented (yellow, top panel) and documented (purple, bottom panel) subgroups. Solid line shows boundary separating region with unequal probability of belonging to documented (below line) and non-documented control (above line).

We identified height z-score as the most influential factor for predicting clinician recognition of ARDS. The probability of a observing a specific pair of values of lowest ${\hat{\text{V}}}_{\text{T}}$ and height z-score for both the pooled documented and control non-documented subgroups is mapped to a heatmap in Fig 3, which includes the line at which the probabilities of the observation being drawn from each of the subgroups (pooled documented vs. control non-documented) were equal (Fig 3, black line). We used this line to estimate whether a patient from the ARDS non-documented subgroup is “recognized” (below the line) or “unrecognized” (above the line). Combining the pooled documented subgroup with the patients from the ARDS non-documented subgroup recognized in this manner yields the following estimates of clinician recognition rates of ARDS for each ARDS severity category: mild, 54%; moderate, 63%; severe, 73% (Table 2).

**Table 2:** Rates of physician recognition of ARDS by hypoxemia severity in LUNG SAFE.

Severity	Documentation	Recognition	
		LUNG SAFE study [6]	Naïve Bayes
	N (%)	% (95% CI)	N (%)
Mild 200 < P_a_O_2_/F_I_O_2_ ≤ 300	73 (19)	51.3 (47.5, 55.5)	206 (54)
Moderate 100 < P_a_O_2_/F_I_O_2_ ≤ 200	301 (24)	65.3 (62.4, 68.1)	800 (63)
Severe P_a_O_2_/F_I_O_2_ ≤ 100	321 (38)	78.5 (74.8, 81.8)	624 (73)

ARDS: acute respiratory distress syndrome.

As Fig 3 illustrates, outside of approximately height z-score±1.0, the separation line is relatively flat at 8mL/kg PBW (z<-1.0) and 6mL/kg PBW (z>1.0), but it decreases linearly for z-scores between -1.0 and +1.0.

For the assist control/volume control (VAC) sensitivity analysis subgroups, documentation and recognition rates are lower than the full cohort; recognition of ARDS was estimated as: mild, 55%; moderate, 68%; severe, 79% (S3 Fig, S8 Table).

## Discussion

Prior to the COVID-19 pandemic, the management of ARDS with evidence-based practices was far from optimal, with studies suggesting a primary barrier of under-recognition of ARDS. [6,9–10] Now, critical care medicine is confronted with COVID-19, which in its severe form frequently presents as ARDS. [1–4] While the mere presence of COVID-19 may improve ARDS recognition, this benefit could be curtailed by the unprecedented changes in critical care structure, staffing, and practice brought about by the pandemic. Consequently, tools that enable widespread ARDS recognition become critical. The goal of this study was to validate an automated EHR-based data-driven tool that can be employed to estimate the ARDS recognition rate of a single clinician, a hospital unit, or even a system of hospitals.

In a prior study of 361 ARDS patients in 9 ICUs, we identified which patient and physician factors were associated with LTVV utilization, and then developed a computational model to estimate ARDS recognition, which found recognition rates that were similar to LUNG SAFE recognition of severe ARDS only. [11] Our current study validates and generalizes these results in a cohort of 2,705 ARDS patients in 459 ICUs, finding that our automated tool accurately replicates LUNG SAFE clinician-reported ARDS recognition across all ARDS severity groups using only patient height, gender, and tidal volume in patients where ARDS is not itself documented. The large and diverse nature of the LUNG SAFE cohort lends powerful support to the generalizability of these conclusions across geographic regions and clinical practice variation.

Confirming prior work, [11] we found that greater height was predictive of lower standardized tidal volumes—which is already adjusted for height—in both ARDS and non-ARDS patients. Remarkably, the patient’s height has a greater influence on their standardized tidal volume than documentation of ARDS. This finding suggests a ventilator management approach that is not driven by recognition of ARDS. Clinicians are likely using a heuristic when delivering tidal volumes, irrespective of ARDS status or recognition: a default tidal volume for most ventilated patients (e.g., 450mL, 500mL), with some adjustment for extremes of height. This explanation is supported by Fig 3. Between height z-score -1.0 to +1.0, where the majority of patients are found, there is a linear decrease in ${\hat{\text{V}}}_{\text{T}}$, exactly what a default tidal volume would produce. Shorter and taller patients have flat ${\hat{\text{V}}}_{\text{T}}$ at approximately 8mL/kg PBW and 6mL/kg PBW, respectively. In this scenario, taller patients receive strict LTVV (6mL/kg PBW)—regardless of ARDS status—by virtue of their height alone.

An alternative possibility—that clinicians intentionally treat all patients with acute hypoxemic respiratory failure similarly, regardless of ARDS status—is unlikely. There is significant evidentiary support for LTVV for ARDS patients (and a lack of such evidence for non-ARDS patients), [7–8] and the association we identified between ARDS documentation and standardized tidal volumes supports some distinction in tidal volume use based on ARDS vs. non-ARDS status.

The relationship between height and tidal volume has important implications for measuring LTVV adherence and therefore the implementation of strategies to improve LTVV utilization. Prior studies exploring barriers to LTVV use in ARDS have used a flat standardized tidal volume threshold (e.g., less than 6.5 mL/kg PBW) to measure LTVV delivery. [9, 14] Our results strongly suggest that this approach is inadequate. Using ${\hat{\text{V}}}_{\text{T}}$ alone as indicative of intentional LTVV use does not account for the additional role that height plays as a driving force behind the tidal volume delivered to a patient. Thus, clinicians may *appear* to be delivering LTVV more often than what is recommended or is truly intended. This practice could mask the impact of important barriers to LTVV implementation, subsequently delaying the design of effective strategies to overcome these barriers.

Our analysis has several limitations. First, there was no stipulation as to whom filled out the CRF for patients at each site. Thus, it is possible that ARDS documentation may not represent a true measure of bedside clinician ARDS recognition. Second, because clinician belief is not thought to be a major barrier to LTVV use for ARDS patients, [12–13] we assumed that if a clinician recognizes ARDS, they would attempt to deliver a lower tidal volume. Third, the LUNG SAFE documentation process involved prompting clinicians about ARDS, rather than relying on *de novo* differential diagnosis generation; therefore, even though LUNG SAFE was a large multinational observational study, it may still not reflect recognition in real world circumstances. Fourth, since the recognition tool builds on documentation of ARDS on both entry and exit CRFs to be considered “recognized,” the 12.2% of patients who developed ARDS after ICU admission could have been incorrectly classified as “unrecognized.” However, the sensitivity analysis we performed using documentation only on the exit CRF, which would have captured patients who developed ARDS after study entry, demonstrated no difference in outcomes. Fifth, this study is restricted to the validation of a previously-developed tool and does not explore whether applying other computational approaches—such as Random Forest or Gradient Boosting—to the task of discriminating recognized versus non-recognized ARDS would yield higher performance. We feel justified in this decision for two reasons: 1) because of the minimal difference between our estimated recognition rates and the original LUNG SAFE recognition rates, and, second, the inherent uncertainty in determining which patients were recognized that prevents us from having access to a perfectly accurate ground-truth. Finally, the LUNG SAFE study represents a pre-COVID understanding of ARDS management. ARDS management, particularly ARDS recognition, may have evolved because of COVID-19.

## Supporting information

S1 Text Clinical characteristics. (DOCX)

S1 Table Data availability for LUNG SAFE full cohort. (DOCX)

S2 Table Data availability for LUNG SAFE VAC subgroup. (DOCX)

S3 TablePredictors of lowest ${\hat{\text{V}}}_{\text{T}}$ (mL/kg PBW) in LUNG SAFE documentation subgroups (β-coefficient [95% CI]).(DOCX)

S4 Table Predictors of lowest standardized tidal volume (mL/kg PBW) in non-documented subgroups (β-coefficient [95% CI]). (DOCX)

S5 Table Covariance analysis in ARDS subgroup (β-coefficient [95% CI]). (DOCX)

S6 Table Multivariable models of lowest standardized tidal volume (mL/kg PBW) in ARDS cohort. (DOCX)

S7 TablePredictors of lowest ${\hat{\text{V}}}_{\text{T}}$ (mL/kg PBW) in VAC subgroup (β-coefficient [95% CI]).(DOCX)

S8 Table Rates of physician recognition of ARDS by hypoxemia severity in LUNG SAFE VAC subgroup. (DOCX)

S1 Fig Naïve Bayes boundary between recognized and unrecognized regions with 95% confidence intervals from bootstrapping–LUNG SAFE cohort. Solid line shows boundary separating region with unequal probability of belonging to documented (below line) and non-documented control (above line) with 95% confidence bands from bootstrapped data (shaded region).
(DOCX)

S2 FigEffects of lowest P_a_O_2_/F_I_O_2_ ratio on standardized tidal volume (${\hat{\text{V}}}_{\text{T}}$) and ARDS documentation in ARDS and control cohorts.Top panels show patients with ARDS documented (purple diamonds) and non-documented patients (tan circles). Gray areas indicate LTVV range from current guidelines, with dashed line at 6.5 mL/kg PBW. Solid lines show linear (${\hat{\text{V}}}_{\text{T}}$) and logistic (documentation) fits for scatter plot data (shaded regions, 95% confidence bands). Reported beta coefficients are for standardized inputs.(DOCX)

S3 Fig Kernel Density Estimation for control non-documented and pooled documented patients in LUNG SAFE (VAC subgroup). Heatmaps of kernel density estimated probability density for data from control non-documented (yellow, top panel) and documented (purple, bottom panel) subgroups. Solid line shows boundary separating region with unequal probability of belonging to documented (below line) and non-documented control (above line). VAC: assist control/volume control mode.
(DOCX)
