# Supplementary material for: Validation of a tool for estimating clinician recognition of ARDS using data from the international LUNG SAFE study
Source: PLOS Digit Health. 2023 Aug 25;2(8):e0000325. doi: 10.1371/journal.pdig.0000325 (PMC10456149; doi:10.1371/journal.pdig.0000325)
Supplement: S1 Text — (DOCX) [file pdig.0000325.s001.docx]

**S1 Text**

*Clinical Characteristics*

Based on previous work, we constructed a multivariable ordinary least squares (OLS) regression using a forward step-wise approach including the following characteristics for both cohorts and all subgroups: P_a_O_2_/F_I_O_2_, plateau pressure (P_plat_, cm H_2_O), patient weight (kg) at ICU admission, and patient height z-score (calculated separately based on patient gender; we chose gender-normalized height z-score because it allows for comparison of male and female patients within the same analysis).[11] Further, we considered the number of chest X-ray quadrants with infiltrates (0-4), sequential organ failure assessment (SOFA) score, geographic region, most frequently used ventilator mode, patient study enrollment age (time difference between the start of patient enrollment at a site and the enrollment of a specific patient at that site), and clinician documentation of ARDS recognition (study entry, study end, and both). For P_a_O_2_/F_I_O_2_, number of chest X-ray quadrants with infiltrates, P_plat_, and SOFA, we extracted their worst value, and their value at study entry and study end. Before performing regression analyses, we linear transformed continuous variables to the range [0, 1].
