# Supplementary material for: Validation of a tool for estimating clinician recognition of ARDS using data from the international LUNG SAFE study
Source: PLOS Digit Health. 2023 Aug 25;2(8):e0000325. doi: 10.1371/journal.pdig.0000325 (PMC10456149; doi:10.1371/journal.pdig.0000325)
Supplement: S1 Table — (DOCX) [file pdig.0000325.s002.docx]

**S1 Table. Data availability for LUNG SAFE full cohort.*^a^***

|  | **ARDS** | | | | **Control** | | | | **Pooled**  **documented** | | |
| --- | --- | --- | --- | --- | --- | --- | --- | --- | --- | --- | --- |
|  | **All** | **Non-documented** | | | **All** | **Non-documented** | | |  |  |  |
| **Factors** |  | **Both** | **End** | **Entry** |  | **Both** | **End** | **Entry** | **Both** | **End** | **Entry** |
| Height | 2584 | 899 | 991 | 1774 | 1193 | 887 | 903 | 1107 | 760 | 1768 | 896 |
| P_a_O_2_/F_I_O_2_ |  |  |  |  |  |  |  |  |  |  |  |
| Entry | 2584 | 899 | 991 | 1774 | 1190 | 885 | 901 | 1104 | 760 | 1767 | 896 |
| End | 1621 | 531 | 594 | 1098 | 642 | 469 | 476 | 597 | 483 | 1124 | 568 |
| Lowest | 2584 | 899 | 991 | 1774 | 1192 | 887 | 903 | 1106 | 760 | 1767 | 896 |
| Documentation |  |  |  |  |  |  |  |  |  |  |  |
| Entry | 2584 | 899 | 991 | 1774 | 1193 | 887 | 903 | 1107 |  |  |  |
| End | 2507 | 899 | 991 | 1718 | 1155 | 887 | 903 | 1076 |  |  |  |
| Both |  |  |  |  |  |  |  |  |  |  |  |
| Chest imaging quadrants |  |  |  |  |  |  |  |  |  |  |  |
| Entry | 2527 | 871 | 961 | 1726 | 1011 | 740 | 754 | 932 | 750 | 1715 | 880 |
| End | 1261 | 412 | 461 | 860 | 496 | 364 | 374 | 453 | 375 | 875 | 444 |
| Highest | 2566 | 889 | 981 | 1761 | 1133 | 840 | 854 | 1052 | 754 | 1754 | 886 |
| SOFA score |  |  |  |  |  |  |  |  |  |  |  |
| Entry | 1493 | 473 | 527 | 1016 | 624 | 461 | 468 | 577 | 449 | 1049 | 524 |
| End | 966 | 338 | 370 | 653 | 373 | 271 | 275 | 343 | 299 | 649 | 343 |
| Highest | 1888 | 601 | 669 | 1266 | 781 | 565 | 573 | 724 | 582 | 1337 | 679 |
| ICU admission weight | 2554 | 888 | 980 | 1750 | 1173 | 875 | 890 | 1090 | 753 | 1743 | 887 |
| Study Age |  | 899 | 991 | 1774 |  | 887 | 903 | 1107 | 760 | 1768 | 896 |
| Region | 2584 |  |  |  | 1193 |  |  |  |  |  |  |
| Modality | 2445 | 818 | 907 | 1664 | 1119 | 824 | 839 | 1037 | 732 | 1705 | 863 |

*^a^* All data is number of patients.[11]
