# Supplementary material for: Validation of a tool for estimating clinician recognition of ARDS using data from the international LUNG SAFE study
Source: PLOS Digit Health. 2023 Aug 25;2(8):e0000325. doi: 10.1371/journal.pdig.0000325 (PMC10456149; doi:10.1371/journal.pdig.0000325)
Supplement: S2 Table — (DOCX) [file pdig.0000325.s003.docx]

**S2 Table. Data availability for LUNG SAFE VAC subgroup.*^a^***

|  | **ARDS** | | | | **Control** | | | | **Pooled**  **documented** | | |
| --- | --- | --- | --- | --- | --- | --- | --- | --- | --- | --- | --- |
|  | **All** | **Non-documented** | | | **All** | **Non-documented** | | |  |  |  |
| **Factors** |  | **Both** | **End** | **Entry** |  | **Both** | **End** | **Entry** | **Both** | **End** | **Entry** |
| Height | 399 | 126 | 132 | 271 | 203 | 154 | 155 | 184 | 139 | 301 | 147 |
| P_a_O_2_/F_I_O_2_ |  |  |  |  |  |  |  |  |  |  |  |
| Entry | 399 | 126 | 132 | 271 | 203 | 154 | 155 | 184 | 139 | 301 | 147 |
| End | 248 | 68 | 73 | 167 | 103 | 74 | 74 | 91 | 87 | 195 | 93 |
| Lowest | 399 | 126 | 132 | 271 | 203 | 154 | 155 | 184 | 139 | 301 | 147 |
| Documentation |  |  |  |  |  |  |  |  |  |  |  |
| Entry | 399 | 126 | 132 | 271 | 203 | 154 | 155 | 184 |  |  |  |
| End | 385 | 126 | 132 | 258 | 203 | 154 | 155 | 184 |  |  |  |
| Both |  |  |  |  |  |  |  |  |  |  |  |
| Chest imaging quadrants |  |  |  |  |  |  |  |  |  |  |  |
| Entry | 393 | 123 | 129 | 265 | 169 | 125 | 126 | 152 | 137 | 293 | 145 |
| End | 215 | 62 | 67 | 145 | 102 | 80 | 81 | 92 | 73 | 160 | 80 |
| Highest | 398 | 125 | 131 | 270 | 191 | 144 | 145 | 173 | 138 | 299 | 146 |
| SOFA score |  |  |  |  |  |  |  |  |  |  |  |
| Entry | 224 | 63 | 69 | 147 | 106 | 74 | 74 | 92 | 84 | 174 | 91 |
| End | 132 | 45 | 49 | 87 | 47 | 33 | 33 | 40 | 47 | 91 | 52 |
| Highest | 260 | 76 | 82 | 172 | 122 | 88 | 88 | 108 | 95 | 200 | 102 |
| ICU admission weight | 396 | 126 | 132 | 270 | 201 | 152 | 153 | 182 | 137 | 298 | 145 |
| Study Age |  | 126 | 132 | 271 |  | 154 | 155 | 184 | 139 | 301 | 147 |
| Region | 399 |  |  |  | 203 |  |  |  |  |  |  |

*^a^* All data is number of patients.[11] VAC: assist control/volume control mode.
