# Supplementary material for: Validation of a tool for estimating clinician recognition of ARDS using data from the international LUNG SAFE study
Source: PLOS Digit Health. 2023 Aug 25;2(8):e0000325. doi: 10.1371/journal.pdig.0000325 (PMC10456149; doi:10.1371/journal.pdig.0000325)
Supplement: S3 Table — (DOCX) [file pdig.0000325.s004.docx]

S3 Table. Predictors of lowest V̂_T_ (mL/kg PBW) in LUNG SAFE documentation subgroups (β-coefficient [95% CI]).[11]

|  | **ARDS non-documented** | | **Control non-documented** | | **Pooled documented** | |
| --- | --- | --- | --- | --- | --- | --- |
| **Factors** | **End** | **Entry** | **End** | **Entry** | **End** | **Entry** |
| Height Z Score | **-3.87*^a^***  **[-4.47, -3.28]** | **-3.72*^a^***  **[-4.24, -3.20]** | **-5.15*^a^***  **[-6.19, -4.12]** | **-6.68*^a^***  **[-7.70, -5.65]** | **-6.42*^a^***  **[-7.16, -5.68]** | **-5.52*^a^***  **[-6.37, -4.68]** |
| P_a_O_2_/F_I_O_2_ |  |  |  |  |  |  |
| Entry | 0.35  [-0.11, 0.81] | 0.52  [0.18, 0.86] | 0.25  [-0.3, 0.81] | 0.18  [-0.3, 0.66] | 0.49  [0.13, 0.85] | 0.74  [0.21, 1.28] |
| End | 0.23  [-0.61, 1.08] | 0.31  [-0.3, 0.91] | -0.65  [-1.73, 0.43] | -0.44  [-1.28, 0.39] | -0.26  [-0.79, 0.27] | -0.46  [-1.29, 0.37] |
| Lowest | 0.87  [0.37, 1.37] | **1.08*^a^***  **[0.69, 1.46]** | 0.46  [-0.16, 1.07] | 0.44  [-0.07, 0.95] | **1.1*^a^***  **[0.69, 1.51]** | **1.46*^a^***  **[0.87, 2.05]** |
| Chest imaging quadrants |  |  |  |  |  |  |
| Entry | -0.03  [-0.32, 0.25] | -0.39  [-0.59, -0.19] | -0.19  [-0.65, 0.26] | -0.34  [-0.71, 0.02] | **-0.89*^a^***  **[-1.17, -0.62]** | **-0.88*^a^***  **[-1.3, -0.46]** |
| End | -0.29  [-0.84, 0.26] | -0.74  [-1.12, -0.35] | -0.43  [-1.2, 0.34] | -0.53  [-1.1, 0.03] | -0.73  [-1.1, -0.37] | -0.66  [-1.22, -0.1] |
| Highest | -0.61  [-1.13, -0.09] | **-1.17*^a^***  **[-1.55, -0.78]** | -0.62  [-1.12, -0.11] | -0.69  [-1.1, -0.29] | **-1.46*^a^***  **[-1.82, -1.09]** | **-1.64*^a^***  **[-2.22, -1.07]** |
| SOFA score |  |  |  |  |  |  |
| Entry | 0.03  [-0.85, 0.9] | 0.07  [-0.61, 0.75] | -0.4  [-1.32, 0.51] | 0.13  [-0.57, 0.83] | 0.49  [-0.11, 1.08] | 0.5  [-0.4, 1.4] |
| End | -0.04  [-0.96, 0.88] | -0.09  [-0.79, 0.6] | 0.35  [-0.72, 1.43] | 0.85  [-0.06, 1.76] | 0.47  [-0.18, 1.12] | 0.52  [-0.36, 1.4] |
| Highest | -0.25  [-1.01, 0.51] | -0.43  [-1.04, 0.18] | -0.36  [-1.32, 0.6] | 0.08  [-0.65, 0.82] | 0.07  [-0.5, 0.64] | 0.18  [-0.65, 1.01] |
| ICU admission weight | -0.33  [-1.26, 0.61] | -0.9  [-1.77, -0.04] | -0.41  [-1.9, 1.07] | -0.52  [-1.78, 0.74] | **-1.9*^a^***  **[-2.81, -0.98]** | -2.21  [-3.64, -0.78] |
| Study Age | -1.26  [-2.6, 0.07] | -0.98  [-2.1, 0.14] | -1.43  [-2.89, 0.03] | -1.56  [-2.8, -0.33] | -0.08  [-0.9, 0.75] | 0.66  [-0.51, 1.82] |
| Modality | 0.26  [-0.23, 0.76] | 0.3  [-0.09, 0.69] | 0.16  [-0.49, 0.81] | -0.04  [-0.58, 0.49] | -0.21  [-0.62, 0.21] | -0.2  [-0.83, 0.43] |

*^a^* *P*<0.00009. Empty cells indicate category was not used due to data being unavailable or not relevant.
