# Supplementary material for: Validation of a tool for estimating clinician recognition of ARDS using data from the international LUNG SAFE study
Source: PLOS Digit Health. 2023 Aug 25;2(8):e0000325. doi: 10.1371/journal.pdig.0000325 (PMC10456149; doi:10.1371/journal.pdig.0000325)
Supplement: S4 Table — (DOCX) [file pdig.0000325.s005.docx]

**S4 Table. Predictors of lowest standardized tidal volume (mL/kg PBW) in non-documented subgroups (β-coefficient [95% CI]).**

| **Factor** | **ARDS non-documented**  **univariable** | **Control non-documented**  **univariable** |
| --- | --- | --- |
| Height Z score | **-3.5*^a^***  **[-4.0, -3.0]** | **-7.7*^a^***  **[-8.7, -6.6]** |
| P_a_O_2_/F_I_O_2_ ratio |  |  |
| Entry | 0.37  [-0.11, 0.85] | 0.25  [-0.27, 0.77] |
| End | 0.35  [-0.57, 1.3] | -0.43  [-1.4, 0.51] |
| Lowest | 0.86  [0.33, 1.4] | 0.43  [-0.14, 1.0] |
| P_plat_ |  |  |
| Entry | -0.95  [-1.8, 0.33] | -0.21  [-1.3, 0.85] |
| End | -0.95  [-2.0, 0.06] | 0.06  [-1.0, 1.2] |
| Highest | -1.1  [-2.1, -0.15] | -0.28  [-1.3, 0.80] |
| Chest imaging quadrants |  |  |
| Entry | 0.0  [-0.30, 0.30] | -0.13  [-0.56, 0.29] |
| End | -0.34  [-0.93, 0.25] | -0.23  [-0.88. 0.43] |
| Highest | -0.51  [-1.1, 0.04] | -0.56  [-1.0, -0.09] |
| SOFA |  |  |
| Entry | 0.11  [-0.85, 1.1] | -0.47  [-1.3, 0.33] |
| End | -0.13  [-1.1, 0.84] | 0.31  [-0.75, 1.4] |
| Highest | -0.18  [-1.0, 0.65] | -0.47  [-1.3, 0.39] |
| ICU admission weight | -0.33  [-1.3, 0.65] | -0.70  [-2.1. 0.70] |
| Study Age | -1.53  [-2.9, -0.16] | -1.6  [-3.0, -0.22] |
| Modality | 0.25  [-0.27, 0.77] | 0.03  [-0.58, 0.65] |

*^a^* *P*<0.00009. Empty cells indicate category was not used due to data being unavailable or not relevant.
