# Supplementary material for: Validation of a tool for estimating clinician recognition of ARDS using data from the international LUNG SAFE study
Source: PLOS Digit Health. 2023 Aug 25;2(8):e0000325. doi: 10.1371/journal.pdig.0000325 (PMC10456149; doi:10.1371/journal.pdig.0000325)
Supplement: S5 Table — (DOCX) [file pdig.0000325.s006.docx]

**S5 Table. Covariance analysis in ARDS subgroup (β-coefficient [95% CI]).**

|  | **CXR quadrants** | | | **P_a_O_2_/F_I_O_2_** | | | **Plateau Pressure** | | **Documentation** | |
| --- | --- | --- | --- | --- | --- | --- | --- | --- | --- | --- |
|  | Beginning | End | Highest | Beginning | End | Lowest | Beginning | Highest | Beginning | End |
| **CXR quadrants** |  |  |  |  |  |  |  |  |  |  |
| Beginning |  | 0.49  [0.42, 0.55] | 0.83  [0.81, 0.85] | -0.43  [-0.5, -0.37] | -0.2  [-0.31, -0.09] | -0.5  [-0.58, -0.43] | 0.65  [0.48, 0.82] | 0.92  [0.73, 1.1] | **0.945*^a^***  **[0.76, 1.1]** | **1.16*^a^***  **[0.97, 1.35]** |
| End |  |  | **0.76*^a^***  **[0.69, 0.82]** |  | -0.67  [-0.76, -0.58] | **-0.33*^a^***  **[-0.41, -0.25]** |  | **0.6*^a^***  **[0.4, 0.8]** | 0.42  [0.07, 0.77] | **1.49*^a^***  **[1.14, 1.85]** |
| Highest |  |  |  |  | -0.14  [-0.19, -0.09] | **-0.3*^a^***  **[-0.33, -0.26]** |  | **0.53*^a^***  **[0.44, 0.62]** | **2.00*^a^***  **[1.61, 2.40]** | **2.67*^a^***  **[2.29, 3.05]** |
| **PaO2/FiO2** |  |  |  |  |  |  |  |  |  |  |
| Beginning |  |  |  |  |  |  | -0.49  [-0.58, -0.4] |  | **-1.25*^a^***  **[-1.59, -0.90]** | **-1.86*^a^***  **[-2.20, -1.53]** |
| End |  |  |  |  |  | 0.52  [0.47, 0.56] |  | -0.38  [-0.47, -0.28] | -0.61  [-1.16, -0.06] | **-2.09*^a^***  **[-2.64, -1.55]** |
| Lowest |  |  |  |  |  |  |  | **-0.62*^a^***  **[-0.7, -0.54]** | **-1.62*^a^***  **[-2.03, -1.21]** | **-2.66*^a^***  **[-3.06, -2.27]** |
| **Plateau Pressure** |  |  |  |  |  |  |  |  |  |  |
| Beginning |  |  |  |  |  |  |  |  | 1.62  [0.78, 2.46] | **3.23*^a^***  **[2.31, 4.14]** |
| Highest |  |  |  |  |  |  |  |  | **1.87*^a^***  **[0.95, 2.80]** | **5.12*^a^***  **[4.05, 6.19]** |

*^a^* *P*<0.00009. Empty cells indicate category was not used due to data being unavailable or not relevant.
