# Supplementary material for: Validation of a tool for estimating clinician recognition of ARDS using data from the international LUNG SAFE study
Source: PLOS Digit Health. 2023 Aug 25;2(8):e0000325. doi: 10.1371/journal.pdig.0000325 (PMC10456149; doi:10.1371/journal.pdig.0000325)
Supplement: S6 Table — (DOCX) [file pdig.0000325.s007.docx]

**S6 Table. Multivariable models of lowest standardized tidal volume (mL/kg PBW) in ARDS cohort.**

| **Model** | **AIC** | **BIC** |
| --- | --- | --- |
| V̂_T_ ~ Height_z | 13190 | 13210 |
| V̂_T_ ~ lowest_PF | 10110 | 10122 |
| V̂_T_ ~ Documentation | 10142 | 10154 |
| V̂_T_ ~ Height_z + lowest_PF | 10211 | 10228 |
| V̂_T_ ~ Height_z + Documentation | 9974 | 9992 |
| **V̂_T_ ~ Height_z + lowest_PF + Documentation*^a^*** | **9922** | **9945** |
| V̂_T_ ~ Height_z + lowest_PF + Documentation + Documentation:lowest_PF | 9924 | 9953 |

*^a^* Model selected as best fit. AIC: Akaike Information Criterion for goodness of fit. BIC: Bayesian Information Criterion for goodness of fit.
