# Supplementary material for: Validation of a tool for estimating clinician recognition of ARDS using data from the international LUNG SAFE study
Source: PLOS Digit Health. 2023 Aug 25;2(8):e0000325. doi: 10.1371/journal.pdig.0000325 (PMC10456149; doi:10.1371/journal.pdig.0000325)
Supplement: S7 Table — (DOCX) [file pdig.0000325.s008.docx]

S7 Table. Predictors of lowest V̂_T_ (mL/kg PBW) in VAC subgroup (β-coefficient [95% CI]).

|  | **ARDS** | **Control** |
| --- | --- | --- |
| Height Z Score | **-3.79*^a^***  **[-4.23, -3.36]** | **-8.14*^a^***  **[-9.61, -6.68]** |
| PaO2/FIO2 |  |  |
| Beginning | 0.92  [0.35, 1.48] | 0.18  [-0.67, 1.02] |
| End | 0.57  [-0.35, 1.49] | -0.7  [-2.34, 0.93] |
| Lowest | 1.01  [0.4, 1.63] | 0.24  [-0.67, 1.14] |
| CXR quadrants |  |  |
| Beginning | -0.36  [-0.68, -0.05] | -0.26  [-0.93, 0.42] |
| End | -1.16  [-1.75, -0.56] | -0.11  [-0.9, 0.68] |
| Highest | -0.81  [-1.28, -0.35] | -0.29  [-1.03, 0.46] |
| SOFA |  |  |
| Beginning | -0.05  [-0.91, 0.81] | -0.9  [-2.27, 0.47] |
| End | -0.38  [-1.42, 0.65] | 0.86  [-0.82, 2.54] |
| Highest | -0.73  [-1.56, 0.1] | -0.56  [-1.74, 0.62] |
| ICU admission weight | -0.42  [-1.5, 0.65] | -0.73  [-2.68, 1.23] |
| Study Age | -0.53  [-1.96,0.89] |  |
| Region | 1.3  [0.04, 2.56] | 2.92  [1.47, 4.37] |

*^a^* *P*<0.00009. Empty cells indicate category was not used due to data being unavailable or not relevant. VAC: assist control/volume control mode.
