# Supplementary material for: Validation of a tool for estimating clinician recognition of ARDS using data from the international LUNG SAFE study
Source: PLOS Digit Health. 2023 Aug 25;2(8):e0000325. doi: 10.1371/journal.pdig.0000325 (PMC10456149; doi:10.1371/journal.pdig.0000325)
Supplement: S8 Table — (DOCX) [file pdig.0000325.s009.docx]

**S8 Table. Rates of physician recognition of ARDS by hypoxemia severity in LUNG SAFE VAC subgroup.**

| **Severity** | **ARDS**  **Documented[11]** | **Recognition** |
| --- | --- | --- |
|  | N (%) | Naïve Bayes, % |
| Mild  200 < P_a_O_2_/F_I_O_2_ ≤ 300 | 18 (29) | 55 |
| Moderate  100 < P_a_O_2_/F_I_O_2_ ≤ 200 | 53 (28) | 68 |
| Severe  P_a_O_2_/F_I_O_2_ ≤ 100 | 68 (46) | 79 |

VAC: assist control/volume control mode.
