# Supplementary material for: Validation of a tool for estimating clinician recognition of ARDS using data from the international LUNG SAFE study
Source: PLOS Digit Health. 2023 Aug 25;2(8):e0000325. doi: 10.1371/journal.pdig.0000325 (PMC10456149; doi:10.1371/journal.pdig.0000325)
Supplement: S1 Fig — Solid line shows boundary separating region with unequal probability of belonging to documented (below line) and non-documented control (above line) with 95% confidence bands from bootstrapped data (shaded region). (DOCX) [file pdig.0000325.s010.docx]

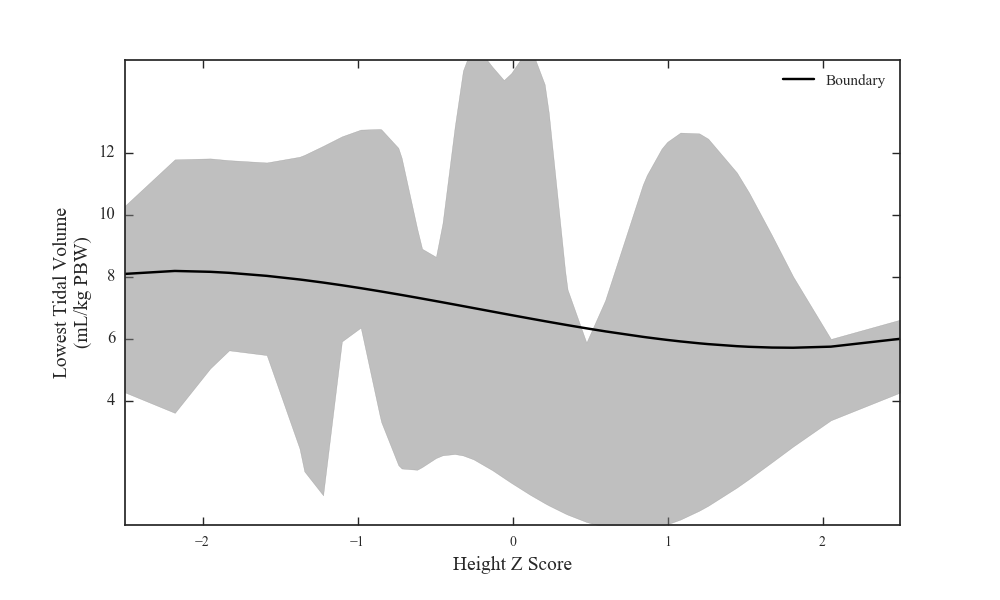


**S1 Fig. Naïve Bayes boundary between recognized and unrecognized regions with 95% confidence intervals from bootstrapping – LUNG SAFE cohort.**

Solid line shows boundary separating region with unequal probability of belonging to documented (below line) and non-documented control (above line) with 95% confidence bands from bootstrapped data (shaded region).
