# Supplementary material for: Validation of a tool for estimating clinician recognition of ARDS using data from the international LUNG SAFE study
Source: PLOS Digit Health. 2023 Aug 25;2(8):e0000325. doi: 10.1371/journal.pdig.0000325 (PMC10456149; doi:10.1371/journal.pdig.0000325)
Supplement: S2 Fig — Top panels show patients with ARDS documented (purple diamonds) and non-documented patients (tan circles). Gray areas indicate LTVV range from current guidelines, with dashed line at 6.5 mL/kg PBW. Solid lines show linear (V^T) and logistic (documentation) fits for scatter plot data (shaded regions, 95% confidence bands). Reported beta coefficients are for standardized inputs. (DOCX) [file pdig.0000325.s011.docx]

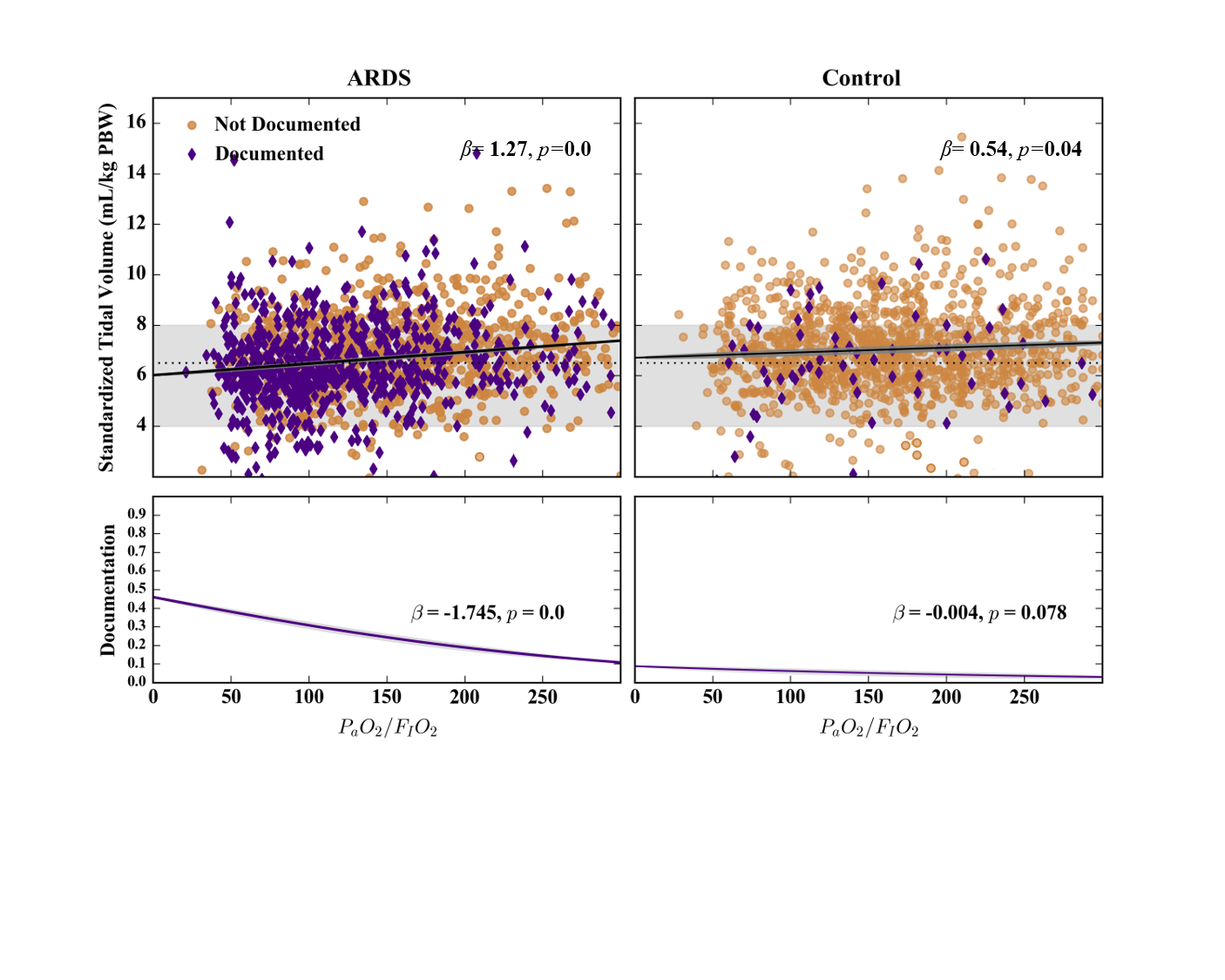


**S2 Fig. Effects of lowest P_a_O_2_/F_I_O_2_ ratio on standardized tidal volume (V̂_T_) and ARDS documentation in ARDS and control cohorts.**

Top panels show patients with ARDS documented (purple diamonds) and non-documented patients (tan circles). Gray areas indicate LTVV range from current guidelines, with dashed line at 6.5 mL/kg PBW. Solid lines show linear (V̂_T_) and logistic (documentation) fits for scatter plot data (shaded regions, 95% confidence bands). Reported beta coefficients are for standardized inputs.
