# Supplementary material for: Validation of a tool for estimating clinician recognition of ARDS using data from the international LUNG SAFE study
Source: PLOS Digit Health. 2023 Aug 25;2(8):e0000325. doi: 10.1371/journal.pdig.0000325 (PMC10456149; doi:10.1371/journal.pdig.0000325)
Supplement: S3 Fig — Heatmaps of kernel density estimated probability density for data from control non-documented (yellow, top panel) and documented (purple, bottom panel) subgroups. Solid line shows boundary separating region with unequal probability of belonging to documented (below line) and non-documented control (above line). VAC: assist control/volume control mode. (DOCX) [file pdig.0000325.s012.docx]

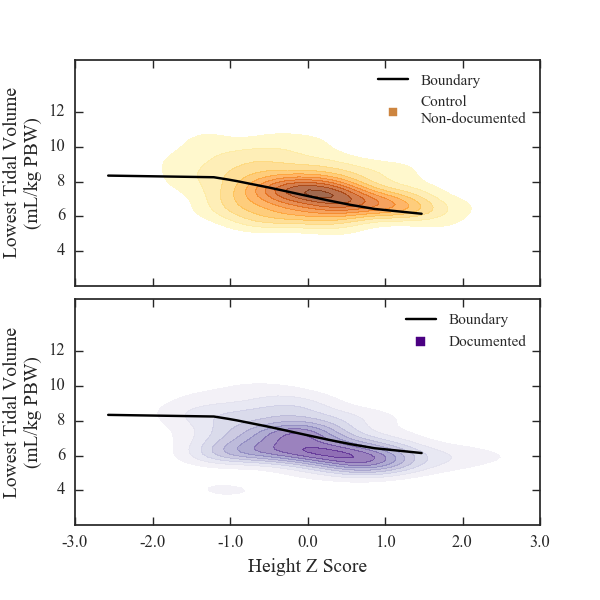


**S3 Fig. Kernel Density Estimation for control non-documented and pooled documented patients in LUNG SAFE (VAC subgroup).**

Heatmaps of kernel density estimated probability density for data from control non-documented (yellow, top panel) and documented (purple, bottom panel) subgroups. Solid line shows boundary separating region with unequal probability of belonging to documented (below line) and non-documented control (above line).

VAC: assist control/volume control mode.
